# Methodological adequacy of articles published in two open-access Brazilian cardiology periodicals

**DOI:** 10.1590/S1516-31802010000200008

**Published:** 2010-03-04

**Authors:** Cristiane Rufino Macedo, Davi Leite da Silva, Maria Eduarda Puga

**Affiliations:** I MSc. Research assistant at the Brazilian Cochrane Center and Doctoral Student in the Discipline of Emergency Medicine and Evidence-Based Medicine, Universidade Federal de São Paulo — Escola Paulista de Medicina (Unifesp-EPM), São Paulo, Brazil.; II Assistant at the Brazilian Cochrane Center.

**Keywords:** Cardiology, Access to information, Epidemiologic research design, Methods, Publications, Cardiologia, Acesso à informação, Projetos de pesquisa epidemiológica, Métodos, Publicações

## Abstract

**CONTEXT AND OBJECTIVE:**

The use of rigorous scientific methods has contributed towards developing scientific articles of excellent methodological quality. This has made it possible to promote their citation and increase the impact factor. Brazilian periodicals have had to adapt to certain quality standards demanded by these indexing organizations, such as the content and the number of original articles published in each issue. This study aimed to evaluate the methodological adequacy of two Brazilian periodicals within the field of cardiology that are indexed in several databases and freely accessible through the Scientific Electronic Library Online (SciELO), and which are now indexed by the Web of Science (Institute for Scientific Information, ISI).

**DESIGN AND SETTING:**

Descriptive study at Brazilian Cochrane Center.

**METHODS:**

All the published articles were evaluated according to merit assessment (content) and form assessment (performance).

**RESULTS:**

Ninety-six percent of the articles analyzed presented study designs that were adequate for answering the objectives.

**CONCLUSIONS:**

These two Brazilian periodicals within the field of cardiology published methodologically adequate articles, since they followed the quality standards. Thus, these periodicals can be considered both for consultation and as vehicles for publishing future articles. For further analyses, it is essential to apply other indicators of scientific activity such as bibliometrics, which evaluates quantitative aspects of the production, dissemination and use of information, and scientometrics, which is also concerned with the development of science policies, within which it is often superimposed on bibliometrics.

## RESUMO

**CONTEXTO E OBJETIVO:**

A utilização de métodos científicos rigorosos tem contribuído para o desenvolvimento de artigos científicos de excelente qualidade metodológica, o que permite a promoção da sua citação, bem como o aumento do fator de impacto. Os periódicos nacionais tiveram que se adequar a alguns padrões de qualidade exigidos pelos indexadores, como por exemplo o conteúdo e o número de artigos originais publicados em cada fascículo. O objetivo foi avaliar a adequação metodológica de dois periódicos brasileiros, na área de cardiologia, indexados em várias bases de dados e disponíveis em livre acesso na Scientific Electronic Library Online (SciELO) e atualmente indexados na Web of Science (Institute for Scientific Information, ISI).

**TIPO DE ESTUDO E LOCAL:**

Estudo descritivo realizado no Centro Cochrane do Brasil.

**MÉTODO:**

Todos os artigos foram avaliados quanto ao mérito (conteúdo) e quanto à forma (desempenho).

**RESULTADO:**

Foi observada uma taxa de 96% de adequação metodológica entre os artigos avaliados, que apresentaram desenho de estudo adequado para responder o objetivo proposto.

**CONCLUSÕES:**

Esses dois periódicos brasileiros na área da cardiologia publicaram artigos metodologicamente adequados, considerando os critérios de qualidade utilizados. Assim, esses periódicos podem ser considerados para consulta e como veículos para a publicação de artigos futuros. Para análises posteriores, é essencial a aplicação de outros indicadores de atividade científica, como a bibliometria, que avalia aspectos quantitativos da produção, disseminação e uso da informação, e a cienciometria, que também considera o desenvolvimento de políticas de ciência, e que muitas vezes se sobrepõe à bibliometria.

## INTRODUCTION

Mankind is now living in the age of information and knowledge, in which people who can master the flow of information that is available are the ones who can keep up to date in some manner and have access to best practices.

It has also been seen that the quantity of the information available in the principal communication media, among which the Internet can be highlighted, leaves people with a sense of anxiety. People sense that, day by day, this avalanche of information has been making them insecure because of the feeling that they are missing something, i.e. some important information that should be coming out at this time. Because of the quantity of data and ease of obtaining it, people feel that they are unable to master it.

In the field of healthcare, there are greater quantities of published information and a better structure for organizing it and making it available, compared with other fields of knowledge. Some of the most important virtual databases (some with free access) containing scientific articles within the field of healthcare are the following:^1^

- Medline (www.pubmed.gov) –– 5,400 indexed periodicals in 37 languages, containing 16 million references to papers, with free access.
- The Cochrane Library (http://cochrane.bvsalud.org/portal/php/index.php?lang=pt) –– with free access in Brazil. This database presents:
  – 6,076 full texts of systematic reviews –– Cochrane Database of Systematic Reviews (CDSR; Cochrane Reviews).
  – 11,887 summaries of non-Cochrane systematic reviews with quality assessment –– Database of Abstracts of Reviews of Effects (DARE; Other Reviews).
  – 608,405 summaries of clinical trials published in Medline, Embase and other sources –– Cochrane Central Register of Controlled Trials (CENTRAL; Clinical Trials).
  – 12,778 methodological studies –– Cochrane Methodology Register (CMR; Methods Studies).
  – 7,724 health technology assessments –– Health Technology Assessment Database (HTA; Technology Assessments).
  – 28,159 economic evaluations –– NHS Economic Evaluation Database (NHSEED; Economic Evaluations)
- Lilacs (Literatura Latino-Americana e do Caribe em Ciências da Saúde - www.bireme.br) –– open access to 500,000 bibliographic records of articles published in around 1,500 health science periodicals.
- Embase (www.embase.com) –– 4,800 indexed periodicals in 30 languages – 12 million article references – access by subscription.

Within the databases cited above, there are some periodicals that form part of a movement known as open access, i.e. provision of open access to the digital medium, in which these periodicals are made available in full text form, free from any onus. The free public availability of these texts on the Internet makes it possible for them to be read, printed and downloaded. In this manner, both the periodical and the paper and its authors will have greater visibility.^2^ This facility of having the full text available, and the complete collections of many periodicals, gives rise to reflection regarding the quality of these periodicals and published studies.

The Institute for Scientific Information (ISI), based in Philadelphia, United States, has assessed scientific production in many fields. The principal parameter used is the so-called impact factor. This is calculated by dividing the number of citations that a periodical receives over a two-year period by the total number of articles published in that periodical.^3^ The use of rigorous scientific methods has contributed towards developing scientific articles of excellent methodological quality. This has made it possible to promote their citation and increase the impact factor.

In Brazil, the impact factor has been used as a parameter for assessing the quality of authors’ and journals’ scientific production, including by government commissions and bodies, such as the Coordination Office for Advancement of University-level Personnel (Coordenação de Aperfeiçoamento de Pessoal de Nível Superior, Capes), which classifies postgraduate programs and their supervisors according to the papers that are published from students’ theses, graded using the impact factors of the journals involved.^4^

Brazilian periodicals have had to adapt to certain quality standards demanded by these indexing organizations, such as the content and the number of original articles published in each issue of the periodical. However, the assessments of researchers’ intellectual production has been limited to the number of citations and the impact factor of the periodicals in which their articles were published, without any specific rigorous critical evaluation of each article. This has led to distortions in science of "publish or perish" nature. There is now a lack of major attention to critical analyses on the general quality of the articles published in each periodical, which would provide a better correlation with the meritocracy of both researchers and periodicals.

## OBJECTIVE

To assess the articles that were published in two Brazilian periodicals in the field of cardiology: Arquivos Brasileiros de Cardiologia and Revista Brasileira de Cirurgia Cardiovascular. These journals are indexed in the most important worldwide databases (PubMed, Lilacs and Scopus) and have recently also gained indexation in the Web of Science/ISI. They are also available in the Scientific Electronic Library (SciELO Online) through open access.

## METHODS

We selected two periodicals within the field of cardiology (Arquivos Brasileiros de Cardiologia and Revista Brasileira de Cirurgia Cardiovascular), which are available through open access (SciELO - Scientific Electronic Library Online - http://www.scielo.org/) and can also be retrieved directly from the databases in which they are indexed (PubMed, Lilacs and Scopus).

The period chosen for analysis was the year 2007, during which the first of these periodicals published two volumes and the second published only one volume.

Two types of evaluation were performed, according to quality standards:^5^

1. Merit assessment (content), consisting of quantitative analysis on all the articles published and their distribution between sections.
2. Form assessment (performance), consisting of analysis on study designs to evaluate how closely the research design matched the study objective.

For the merit assessment, the division of content was investigated. In other words, all the articles were classified with regard to the particular naming of sections in each periodical. No exclusion criteria were determined for this outcome.

To assess form with regard to performance, each article was classified according to its study design, for example: systematic review of the literature, narrative review of the literature, clinical trial, retrospective cohort, prospective cohort, case-control study, case series, cross-sectional study, analytical cross-sectional study, diagnostic test study, guideline study, study on animals, anatomopathological or *in vitro* study, or specialists’ opinion.^6^ Articles named in the journal sections as letter to the editor, editorials or *in memoriam* were excluded from this analysis.

The articles included in the study design analysis were assessed regarding the adequacy of the match between the research design and the study objective. Papers that presented a study design that is recommended for answering the objective were considered to have adequate designs. For example, articles on therapeutics require randomized trials, or systematic reviews or meta-analyses on such trials. Articles on diagnostic tests require accuracy studies, while investigations on the natural history of diseases require cohort studies, and so on.

Two reviewers assessed each article, independently, and the disagreements between them were solved by reaching a consensus.

## RESULTS

All the articles published in the year 2007 in the periodicals Arquivos Brasileiros de Cardiologia and Revista Brasileira de Cirurgia Cardiovascular were evaluated. In Arquivos Brasileiros de Cardiologia, 244 published articles distributed in two volumes (88 and 89) consisting of six issues each (numbered 1 to 6) were found. Volume 88 also presented two supplementary issues (supplement 1 and supplement 2). In Revista Brasileira de Cirurgia Cardiovascular, 90 published articles were distributed in a single volume (volume 22) consisting of four issues (numbered 1 to 4).

Each of the periodicals had sections that were named in a particular way to present its articles. The naming of the sections and the distribution of the articles are presented separately for each periodical in **Tables 1 and 2.**

**Table 1.** Distribution of the articles published in Arquivos Brasileiros de Cardiologia, according to section names

Sections	n
Editorials	1
Original articles	146
Anatomopathological session	6
Clinical radiological session	6
Brief comments	6
Review articles	6
In memoriam	1
Letters to the editor	4
Case reports	24
Points of view	20
Images	11
Clinical updates	7
Guidelines	6
Total	244

**Table 2.** Distribution of the articles published in Revista Brasileira de Cirurgia Cardiovascular, according to section names

Sections	n
Editorials	9
Special articles	2
Original articles	39
Review articles	5
Experiences of the service	4
Case reports	17
Brief communications	1
Clinical surgical correlations	9
Letters to the editor	3
Total	90

With regard to study design, it was found that both periodicals presented different types of study: diagnostic test, prospective cohort, retrospective cohort, case report, case series, clinical trial, narrative review of the literature, cross-sectional prevalence study, analytical cross-sectional study, guidelines, specialists’ opinions and *in vitro* anatomopathological studies, both on animals and on humans.

The articles were evaluated regarding their methodological adequacy, i.e. whether the study design was adequate for the study objective. Ninety-six percent of the articles analyzed, in both periodicals, presented study designs that were adequate for answering the objectives.

## DISCUSSION

In this study, the articles were evaluated according to the section names used in each of the periodicals, along with the study design and its methodological adequacy.

In the merit assessment, we found that each periodical had its own criteria for naming the journal sections. Distributing articles into different sections has the aim of organizing them to facilitate understanding among readers. The two periodicals had some sections in common, such as Editorial, Original Articles, Review Articles, Case Reports and Letters to the Editor, but in different proportions: for example, Arquivos Brasileiros de Cardiologia only published one editorial.^7^ However, there were also some sections that were peculiar to each journal. For example, Arquivos Brasileiros de Cardiologia presented the following sections: Anatomopathological Session (*sic*), Clinical Radiological Session (*sic*), Brief Comments, In Memoriam, Point of View, Image, Clinical Update and Guideline.^8-11^ In Revista Brasileira de Cirurgia Cardiovascular, different sections were found, for example: Special Article, Brief Communication, Clinical Surgical Correlation and Experience of the Service.^12-14^

Among the articles appearing in sections with different names in Arquivos Brasileiros de Cardiologia, there were two in the Clinical-Radiographic Correlation section that in reality were case reports.^8,9^ In the Brief Comments, one cohort study was found.^11^ One of the strong points of the present study was that it made comparisons between recent, simultaneously published journals within the same field, in the same country, without apparent conflicts of interest.

The articles were then classified according to their study design. Cross-sectional studies^15-18^ and case reports were the types most frequently found, respectively in Arquivos Brasileiros de Cardiologia and Revista Brasileira de Cirurgia Cardiovascular.^19,20^

The two periodicals together published a total of five guidelines, both in regular issues and in supplements, as can be seen in Arquivos Brasileiros de Cardiologia.^10,21-24^

Analysis on the articles published according to levels of evidence (Oxford) showed that none of them presented level 1 evidence (systematic reviews), but that there were narrative reviews of the literature. On the other hand, we found studies presenting level 2 evidence, such as clinical trials. Other types of study, such as cohorts, case series, case-control studies, specialists’ opinions and studies on animals were also found.^11,25-47^

A high proportion of the articles in both periodicals (96%) presented methodological adequacy, i.e. a good match between the study objectives and the study design used. This is of great value for increasing the quality of the scientific knowledge produced in the field of cardiology in Brazil. In comparison, in a recent study evaluating papers published on diseases of the larynx, it was found that the study design matched the study objectives in only 3% of them.^48^

## CONCLUSIONS

1. Implications for practice
  From analysis on these two Brazilian periodicals within the field of cardiology, which are indexed in several databases and are available with open access, we found that both of them were methodologically adequate, since they followed the quality standards. Thus, these periodicals can be recommended both for consultation and as vehicles for publishing future articles.
2. Implications for further research
  For future analyses, it is essential to apply other indicators of scientific activity such as bibliometrics, which evaluates quantitative aspects of the production, dissemination and use of information, and scientometrics, which not only evaluates the quantitative aspects of scientific activities but also is concerned with the development of science policies, within which it is often superimposed on bibliometrics.
